# Using Next-Generation Sequencing Transcriptomics To Determine Markers of Post-traumatic Symptoms: Preliminary Findings from a Post-deployment Cohort of Soldiers

**DOI:** 10.1534/g3.118.200516

**Published:** 2019-01-08

**Authors:** Cathy Boscarino, Thomas Nalpathamkalam, Giovanna Pellecchia, Weili Li, Bhooma Thiruvahindrapuram, Daniele Merico

**Affiliations:** * Operational Health and Performance Section, Defence Research & Development Canada - Toronto Research Center, Toronto ON Canada; † The Centre for Applied Genomics, The Hospital for Sick Children, Peter Gilgan Centre for Research and Learning, Toronto ON Canada; ‡ Deep Genomics Inc., Toronto ON Canada

**Keywords:** Post-traumatic stress, expression profiling, next-generation sequencing, soldiers

## Abstract

Post-traumatic stress disorder is a concerning psychobehavioral disorder thought to emerge from the complex interaction between genetic and environmental factors. For soldiers exposed to combat, the risk of developing this disorder is twofold and diagnosis is often late, when much sequela has set in. To be able to identify and diagnose in advance those at “risk” of developing post-traumatic stress disorder, would greatly taper the gap between late sequelae and treatment. Therefore, this study sought to determine whether the transcriptome can be used to track the development of post-traumatic stress disorder in this unique and susceptible cohort of individuals. Gene expression levels in peripheral blood samples from 85 Canadian infantry soldiers (n = 58 participants negative for symptoms of post-traumatic stress disorder and n = 27 participants with symptoms of post-traumatic stress disorder) following return from deployment to Afghanistan were determined using RNA sequencing technology. Count-based gene expression quantification, normalization and differential analysis (with thorough correction for confounders) revealed genes associated to PTSD; *LRP8* and *GOLM1*. These preliminary results provide a proof-of-principle for the diagnostic utility of blood-based gene expression profiles for tracking symptoms of post-traumatic stress disorder in soldiers returning from tour. It is also the first to report transcriptome-wide expression profiles alongside a post-traumatic symptom checklist.

The intensity and frequency of combat seen in Afghanistan sparked a rise in the rate of post-traumatic stress disorder (PTSD) among members of the Canadian Armed Forces (CAF); nearly doubling from 2.8% in 2002 to 5.3% in 2013 (Canadian Forces Mental Health Survey 2013) (Hellmuth *et al.* 2012). This concerning mental health disorder is thought to emerge from complex interactions among multiple genetic and environmental factors. While combat exposure is a risk factor for the development of post-deployment symptoms of PTSD, one’s psychiatric response to combat as a stressor varies considerably between individuals (Wald *et al.* 2013; Breen *et al.* 2015), ranging in spectrum from mild to severe. The ability to identify those at risk of developing PTSD in advance, would greatly taper the gap between late sequelae and treatment. While great advances have been made in our understanding, assessment and treatment of PTSD, the biological etiology remains poorly understood. More specifically, precise biological indicators have yet to be successfully outlined and validated (Tylee *et al.* 2015; Zannas *et al.* 2015).

To address the biological factors underlying this complex psychological disorder, we have taken a genomics-informed approach and aim to determine whether symptoms of PTSD among troops immediately returning from tour are correlated with (and thus could be predicted by) changes in peripheral white blood cell gene expression. Symptoms of PTSD were measured using the military version of the posttraumatic stress disorder Checklist, (PCL), which is traditionally one of the most widely used self-report measures of PTSD and thus extensively used in the military (Dickstein *et al.* 2015; Bovin *et al.* 2016). PCL scores have repeatedly been show to correlate highly with total scores from the diagnostic gold standard – the Clinical- Administered PTSD Scale (CAPS), with scores over 34 as being optimally efficient at diagnosing PTSD (Bovin *et al.* 2016). In our study, we adopted an unbiased approach and profiled the whole human transcriptome using RNA-sequence (RNA- seq), as opposed to the targeted approach adopted in the majority of PTSD studies (Breen *et al.* 2015). Together with the PCL-M, the aim was to determine unique expression patterns associated with symptoms of PTSD in CAF soldiers within one-year following return from tour. This pilot study is the first to report transcriptome-wide expression profiles alongside a PTSD symptom checklist.

## Materials and Methods

The study was approved by the Human Research Ethics Committee (HREC) of Defense Research and Development Canada (DRDC) and the Surgeon General of the Canadian Forces Health Services (CFHS). Permission to access troops was granted by the Chief of Land Operations (COS Land Ops).

### Participants

Canadian infantry soldiers returning from tour in Afghanistan were recruited for participation. Participants were briefed on the study’s protocol and written informed consent was obtained from those who volunteered to participate. Participants remained anonymous for this study by creating a personal identification number (PIN) and any personal information linking the identity of an individual to the PIN was not collected.

### Design and Measures

Soldiers were asked to participate immediately following their return from deployment and every 4 months following that for up to 1-year. Upon enrollment soldiers were asked to complete the following series of questionnaires: a demographic information sheet, the Combat Exposure Scale from the *Deployment risk and Resilience Inventory (DRRI)* (Vogt *et al.* 2013) and the Posttraumatic Stress Disorder Checklist for military personnel (PCL-M) (McDonald and Calhoun 2010). Following completion of the forms,venipuncture was performed by a certified phlebotomist and 2.5 ml of blood was collected using the PAXgene blood RNA collection protocol (PreAnalytiX GmbH, QIAGEN or BD) for gene expression and 4 ml of blood was collected for a complete blood count (CBC). At subsequent data collection periods, participants were asked to complete the PCL-M only and provide another 6.5 ml blood sample. Blood collected for CBC was analyzed by LifeLabs. Blood samples collected for gene expression were stored at -80° in a secured medical specimen freezer located within the Defense Research and Development Canada - Toronto Research Center until further analysis.

### RNA-seq: data generation and bioinformatics

Ribonucleic acid (RNA) was isolated from peripheral blood leukocytes using QIAsymphony PAXgene Blood RNA Kit (Qiagen) and sequenced using the Illumina Hi-Seq 2500. Quality of total RNA samples was assessed on an Agilent Bioanalyzer 2100 RNA Nano chip following Agilent Technologies’ recommendation. Concentration was measured by Qubit RNA HS Assay on a Qubit fluorometer (ThermoFisher). RNA library preparation was performed following the Illumina TruSeq RNA Library Preparation protocol. Briefly, 1000 ng of total RNA was used as the input material and enriched for poly-A mRNA, fragmented by heat and converted to double stranded cDNA; cDNA was end-repaired and adenylated at the 3′ end to create an overhang A to allow for ligation of Illumina adapters with an overhang T; library fragments were amplified under the following conditions: initial denaturation at 98° for 30 sec, followed by 15 cycles of 98° for 10 sec, 60° for 30 sec and 72° for 30 sec, and finally an extension step for 5 min at 72°; Each sample was prepared with different indexed adapters to allow for multiplex sequencing. One microliter (ul) of the RNA libraries was loaded on a Bioanalyzer 2100 DNA High Sensitivity chip (Agilent Technologies) to check for size; RNA libraries were quantified by qPCR using the Kapa Library Quantification Illumina/ABI Prism Kit protocol (KAPA Biosystems). Libraries were pooled in equimolar quantities and paired-end sequenced on a Rapid Run Mode flowcell with the V3 sequencing chemistry on an Illumina HiSeq 2500 platform following Illumina’s recommended protocol to generate paired-end reads of 100-bases in length.

RNA-seq reads were trimmed for low quality read ends and adapter contamination using ‘Trim Galore!’ version 0.3.7 (http://www.bioinformatics.babraham.ac.uk/projects/trim_galore/); specifically, parameters were adjusted to trim low-quality ends from reads (Phred score <20), remove 1bp from 3′ end and retain only reads that have a valid pair after processing. Trimmed reads were also screened against human ribosomal and mitochondrial DNA sequences using fastq_screen version 0.4.4 (https://www.bioinformatics.babraham.ac.uk/projects/fastq_screen/). Finally, RSeQC version 2.6.1 was used to calculate percentage coding exonic reads, estimated insert size and standard deviation (Wang *et al.* 2012).

Quality control metrics for trimmed reads were carried out using FastQC version 0.11.2 (http://www.bioinformatics.babraham.ac.uk/projects/fastqc/), including read percentage GC content and de-duplicated read percentage. RNA-seq reads were subsequently aligned to the human genome reference hg19 (Feb. 2009 assembly of the human genome hg19,GRCh37 Genome Reference Consortium Human Reference 37 (GCA_000001405.1) downloaded from UCSC (http://hgdownload.soe.ucsc.edu/goldenPath/hg19/database/) on June 02, 2014) using the splicing-aware alignment TopHat version 2.0.13 (Trapnell *et al.* 2009) and RefSeq gene models (downloaded in May 2014 from the Illumina iGenome resource: http://support.illumina.com/sequencing/sequencing_software/igenome.html); BAM files (accepted_hits.bam) generated by TopHat were sorted using samtools version 1.1 (http://www.htslib.org/) for downstream analysis. Reads were then counted using HTseq version 0.5.4p1 using the ’intersection-nonempty’ option (Anders *et al.* 2015). Gene expression data are available at GEO with the accession number: GSE109409.

### Statistical analyses of gene expression

This section describes (1) the procedure followed to select technical and biological covariates, (2) the differential gene expression analysis performed using one measurement from each participants, with (a) continuous PCL-M score and (b) dichotomized PCL-M score, and (3) differential gene expression analysis utilizing all gene expression values from each participant.

#### Selection of Covariates:

Global gene expression patterns captured by PCA principal components are typically shaped by two main category of factors: (a) meaningful biological variation, in this case PTSD; (b) biological, experimental and technical confounders. Here we used PCA to determine which biological, experimental and technical confounders have a significant contribution to global gene expression patterns, to avoid fitting a generalized linear model for differential expression with an unnecessary large number of covariates, which would be detrimental for the statistical power of the differential expression analysis. To avoid biased results caused by confounders, the following list of 24 covariates was evaluated in order to select a smaller number of covariates with low mutual correlation while highly correlated to gene expression principal components: (A) several covariates representing different study design and sample collection batches were aggregated into a single categorical batch covariate (task force of origin, blood harvesting time during the year, cohort, cohort deployment time, post-deployment time and phase); (B) biological and demographic covariates: age, sex, white blood cell count, lymphocyte count and percentage, neutrophil count and percentage; (C) RNA-seq QC-related covariates: sequencing batches (2 covariates), mean of the read insert size, standard deviation of the read insert size, read percentage GC content, number and percentage of reads lost after trimming, number and percentage of mapped reads, number and percentage of concordantly mapped reads, de- duplicated read percentage, percentage of reads not mapping to human mitochondrial or ribosomal sequences, percentage reads not exonic (as calculated by HTseq), percentage of reads mapping to coding exonic sequence (as calculated by RSeqQC). Read counts were normalized in DESeq version 1.18.0 (Anders and Huber 2010) for between-sample differences. Here, DESeq was used instead of edgeR (Robinson *et al.* 2010) for the convenience of exporting normalized reads. Principal Component Analysis (PCA) was performed using the R ‘prcomp’ function to select the most informative principal components (PCs). We compared the eigenvalues obtained for the real normalized count matrix to the eigenvalues obtained for 5 normalized count matrices after independent permutation of each gene expression vector (corresponding to a matrix row), resulting in the selection of the first 10 PCs. The most informative covariates were then selected using a greedy stepdown linear regression analysis. More in detail, (1) for each covariate, the correlation between the covariate and each PC was tested using a two-sided Mann-Whitney- Wilcoxon test for binary covariates, a Spearman correlation test for continuous covariates, or ANOVA for categorical covariates; only covariates with nominal p-value < 0.1 were selected for the following analysis step. Then (2), for each PC, an incremental linear regression model was constructed by stepwise addition of significantly-correlated covariates (sorted by nominal p- value); only covariates passing a model comparison test (log-likelihood test implemented in R anova.glm, nominal p-value < 0.001, corresponding to a false discovery rate of 2%) were selected. This stepdown procedure ensures that, if a highly predictive variable is already present, any correlated variable added afterward will not be significant and thus will not be selected. Covariates significant for different PCs but highly correlated were pruned in a final step. The PCL-M score did not correlate with any of the top 10 PCs (p value > 0.1). The full list of covariates and their correlation to each PC can be seen in Supplementary Data Set 1.

### Differential gene expression analysis without repeated measurements

Since modeling the presence of multiple correlated measurements per subject is not possible using state-of-the-art statistical tools for RNA-seq differential analysis that include a variance shrinkage model (*e.g.*, edgeR, DESeq, DESeq2 (Love *et al.* 2014)), the first statistical analysis was performed in edgeR after selecting, for each subject, the measurement with the highest PCL-M score (thus reducing the number of samples from 118 to 85). For differential gene expression analysis, we first modeled PCL-M score as a continuous variable. We then analyzed the discretized PCL-M score, defining PCL-M score < 34 as the control group and PCL-M score ≥ 34 as the symptoms group. Gene expression results are reported for both analysis models in the Supplementary Data Set 2.

Read counts were imported after removing genes with low counts (for each gene, we calculated the 75^th^ percentile of raw counts across samples; then we calculated the quantile corresponding to the 35% of that distribution, q35; finally, we discarded genes whose raw count 75^th^ percentile was lower than q35); note that removing genes with low expression levels is recommended for edgeR because these genes can be handled improperly by the model (https://www.bioconductor.org/packages/devel/bioc/vignettes/edgeR/inst/doc/edgeRUsersGuide.pdf). This filtering step resulted in 16,913 genes, from an initial set of 25,369 genes. Sample- wise normalization factors were estimated using the TMM methods as provided in edgeR v3.8.6 and differential analysis was performed also in edgeR v3.8.6 treating each covariate as a block covariate; Reads Per Kilobase per mapped Million reads (RPKMs) were also generated in edgeR v3.8.6 (for data exploration purposes only); finally, differential p-value multiple test correction was performed using the Benjamini-Hochberg False Discovery Rate (BH-FDR) as provided in edgeR v3.8.6.

### Differential gene expression analysis with repeated measurements

To ensure that selecting one measurement corresponding to the highest PCL-M score from each subject does not lead to false positive results, we repeated the association analyses utilizing all repeated measurements from each subject. Specifically, we analyzed 111 out of the 118 samples (7 measurements were excluded due to missing data in one of the covariates). We used the R package ‘glmmADMB’ to fit a Generalized Linear Mixed Model (GLMM), assuming the counts follow a Negative Binomial distribution. A random intercept was included in the model to account for the correction among multiple measurements from the same subject. The counts were regressed on the continuous PCL-M scores, while accounting for selected covariates. We only analyzed a subset of 400 genes that were statistically significant at the 0.05 level from the EdgeR analysis with one measurement per subject. Substantial difference in p-values is only observed at LINC00893 (Supplement Figure 1).

### Other statistical analyses

Demographic and questionnaire data were statistically analyzed using IBM SPSS Statistics V23.0. Statistical significance was set to *P* < 0.05 (2-sided) for all analyses. Mean and standard deviation (SD) was calculated for all characteristics. Group differences were tested using a student’s *t*-test.

### Data Availability

The research protocol was accepted under the Human Research Ethics Committee (HREC) of Defense Research and Development Canada (DRDC) - Protocol 2017-019. Gene expression data are available at GEO with the accession number: GSE109409. Supplementary data sets 1 and 2 can be found on figshare. Supplemental material available at Figshare: https://doi.org/10.25387/g3.7501751.

## Results

Eighty-five (n = 85) male infantry soldiers, with an average age of 29.8 ± 7.4 years, were sampled within one-year following their return from deployment to Afghanistan. When the participants were grouped based on a dichotomized PCL-M score, 58 scored < 34 (Control) and 27 scored ≥ 34 (Symptoms of PTSD), without significant age differences between the two groups (Table 1). Furthermore, the same number of soldiers had a previous deployment as those whose first deployment was Afghanistan both within and between groups (Table 1). Based on the entire cohort, 62.4% were involved in combat of which 38.8% were in the Control group and 23.5% were in the Symptoms group (Table 1). It is worth noting that the Symptoms of PTSD group demonstrated a trend (*P* = 0.08) in reporting higher combat scores compared to the Control group (Table 1); 8.1 ± 2.7 *vs.* 6.64 ± 3.1, respectively.

**Table 1:** Demographic, Clinical and Experiential Characteristics of Canadian Armed Forces Soldiers Returning from Deployment in Afghanistan (n = 85 participants from Task Force 1-10 and Task Force 1-11 - 58 provided one sample, 21 provided two samples and 6 provided three samples, therefore n = 118 samples total). P-value < 0.05 is considered significant

	Control	Symptoms of PTSD	P-value
	(< 34)	(≥ 34 PCL-M)	
Sample size (n/85)	58	27	n/a
Age:	30.3 ± 6.9	28.67 ± 8.5	0.53
Previous Deployment:	(29) 50%	(14) 51.9%	
Afghanistan Only:	(29) 50%	(13) 48.1%	
Combat (%)//DRRI Score:	38.8%// 6.64 ± 3.1	23.5%// 8.1 ± 2.7	0.08
PCL-M Score	22.73 ± 4.46	47.18 ± 10.31*	< 0.01

Peripheral white blood cell gene expression levels were determined using RNA-seq and covariates including multiple measurements with respect to post-deployment time, for a total of 118 samples. More specifically, out of the 85 participants: 58 provided one sample, 21 provided two samples and 6 provided three samples. To control for batch effects, biological and technical confounders, we considered an initial set of 24 covariates and then selected a subset of 7 covariates significantly correlated to gene expression, using a greedy step-down regression procedure combined with normalized gene count Principal Component Analysis (PCA), as described in the methods section. The final set of 7 covariates included aggregate batch, neutrophil count, white blood cell count, read percentage GC content, percentage of mapped reads, percentage reads not exonic and de-duplicated read percentage.

**Figure 1:**
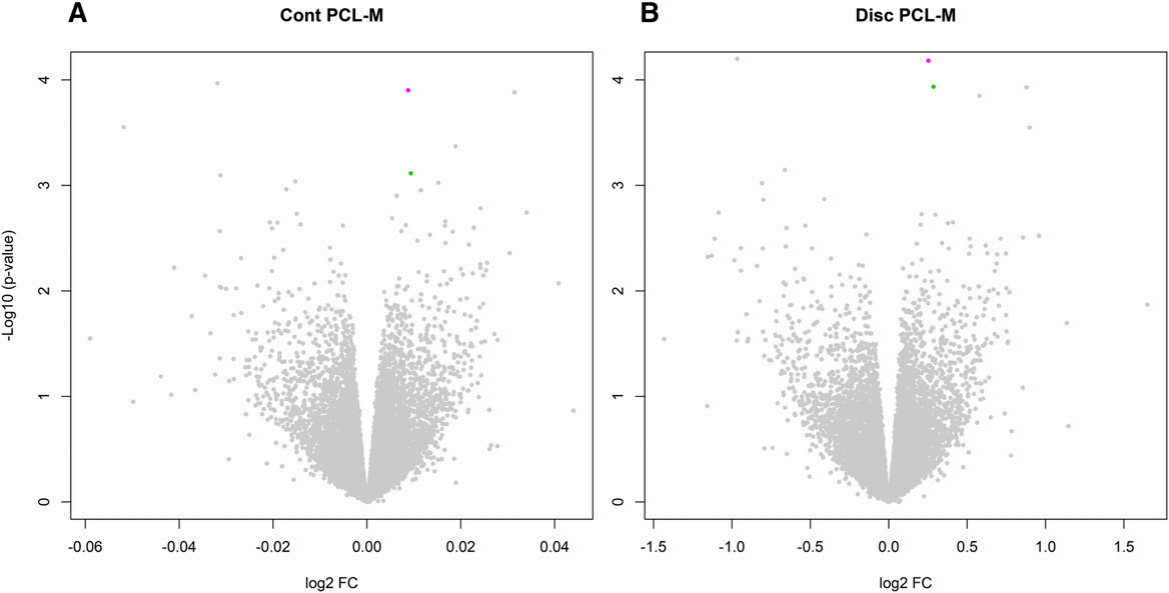
Volcano plots displaying log2 fold-change (log2 FC and – Log10 (p-value) for the continuous (CONT) and discrete (DISC) model; LRP8 depicted in magenta and GOLM1 is depicted in green, whereas all other genes are depicted in gray.

The edgeR discretized model resulted in more significant differential expression: *GOLM1*, *CYP2C8*, *LINC00943* and *LOC100132215* had BH-FDR < 50%; in contrast, for the continuous model only *LRP8* and *LINC00943* had FDR < 75%. The top 5 genes in Table 2 remain associated when all repeated measurements were analyzed using GLMM, with consistent directions of association as detected in the edgeR analysis (Refer to Supplementary Data Set 2). Since these FDRs were very high, we attempted at restricting the number of tests by considering only genes already implicated in behavior and nervous system abnormalities. In particular, we utilized a list of 3,764 human homologs of mouse genes with an established neurobehavioral/neurodevelopmental phenotype (“all neuro”); of these, 2,602 had a neurobehavioral phenotype (“neurobehav”). These genes have already been demonstrated to bear a significantly higher burden of rare copy number losses in neuropsychiatric disorders such as autism and schizophrenia (Pinto *et al.* 2014; Engchuan *et al.* 2015; CNV Schizophrenia Working Group 2017; Marshall *et al*. 2017). Only *LRP8* and *GOLM1* were included in “all neuro”, and when restricting the FDR calculation to these genes, their FDRs improved (17% for the discrete model; 37% and 92% for the continuous model); the FDRs further improved when restricting to “neurobehav” (12% for the discrete model; 25% and 63% for the continuous model). It is also worth noting that the *LRP8* effect size is modest: the symptoms-control expression ratio is ∼1.2; more in detail, 2/27 participants with PCLM >= 34 have LRP8 rpkm > 1, whereas none of the controls does; 6/27 participants with PCLM >= 34 have LRP8 rpkm > 0.9, whereas 4/58 controls do (see Supplementary Data Set 2 for details). Volcano plots for differential expression can be visualized in Figure 1.

**Table 2:** Benjamini-Hochberg-False Discovery Rate (BH-FDR) and Fold-Change (FC) for the top genes significantly dysregulated (with nominal *P* < 0.01) in the EdgeR non- repeated measures analysis in Peripheral Blood Mononuclear Cells at Post-Deployment from Soldiers Returning from Tour in Afghanistan within one-year. *DISC = discretized model; CONT = continuous model

Gene Symbol	Entrez Gene ID	edgeR disc/cont	edgeR FC	edgeR P-value	edgeR BH-FDR all genes	edgeR BH-FDR all neuro	edgeR BH-FDR neurobeh av
LRP8	7804	DISC	1.19	6.58E-05	48%	17%	12%
		CONT	1.01	1.25E-04	74%	37%	25%
GOLM1	51280	DISC	1.22	1.16E-04	48%	17%	12%
		CONT	1.01	7.68E-04	100%	92%	63%
CYP2C8	1558	DISC	0.51	6.32E-05	48%	NA	NA
		CONT	0.98	8.04E-04	100%	NA	NA
LINC00943	100507206	DISC	1.84	1.18E-04	48%	NA	NA
		CONT	1.02	1.31E-04	74%	NA	NA
LOC1001322 15	100132215	DISC	1.49	1.42E-04	48%	NA	NA
		CONT	1.01	4.26E-04	100%	NA	NA

## Discussion

The present study is the first to decipher the relationship between gene expression and symptoms of PTSD in soldiers returning from tour in Afghanistan within one-year post- deployment. Our results are consistent with the pathogenesis of PTSD and support the diagnostic value of gene expression signatures in PBMCs for the development of PTSD phenotype. RNASeq is able to offer unparalleled insight into the molecular causes of many complex diseases such as PTSD and yet, only a limited number of studies using RNASeq on human PTSD samples exist. Using this platform, we sought to investigate whether gene expression signatures from the peripheral blood of soldiers following a tour in Afghanistan are informative of the development of PTSD. Given that this mental health disorder stems from a neuronal abnormality involving three principal brain regions (the amygdala, hippocampus and medial prefrontal cortex) (Girgenti and Duman 2018; Zimmerman *et al.* 2012), brain tissue would be the ideal study material of choice. However, due to the inherent inability to extract brain tissue from live patients, this research area is reserved solely to post-mortem studies. Fortunately, the use of peripheral blood as a successful surrogate to study expression profiles in complex mental health disorders such as PTSD (Segman *et al.* 2005), have been confirmed both in animal models of PTSD as well as post-mortem studies of PTSD brain tissue (Licznerski *et al.* 2015). In fact, a growing body of evidence suggests peripheral blood may in fact reflect dynamic transcription changes in the brain, including PTSD, conferring disease vulnerability (Tylee *et al.* 2015; Girgenti and Duman 2018; Liew *et al.* 2006; De Boever *et al.* 2014). For example, gene association studies have identified four SNPs in the FKBP5 gene as predictors of adult PTSD onset, which have been reflected in a transcriptomic study in post-mortem brain tissue of PTSD patients (Girgenti and Duman 2018). Furthermore, it has been shown that the pro-inflammatory cytokines associated with PTSD, penetrate the blood-brain barrier and induce the expression of genes located within the hippocampus and amygdala (Zimmerman *et al.* 2012). Many studies continue to support the use of peripheral blood for transcriptome research in mental health disorders, and thus we are confident to use PBMCs as our material of choice.

To measure risk of PTSD, we used the PTSD Checklist Military Version (PCL-M) (Dickstein *et al.* 2015; Bovin *et al.* 2016; Hoge *et al.* 2014). Risk is determined by the frequency and severity defined by the Diagnostic and Statistical manual of Mental Disorders (DSM-IV) over the course of the past month. Without a clinical interview, the PCL-M is only suggestive of risk of PTSD, however scores have been shown to strongly correlate with diagnosis. Global scores range from 17 – 85 and while a cut-off point of 50 has been traditionally used for risk of meeting full diagnostic criteria for combat veterans (Forbes *et al.* 2001), a cut-off score of 39 (Dickstein *et al.* 2015) was found to be optimally efficient at identifying full PTSD. Furthermore, scores between 35 and 49 have been shown to classify as risk for meeting subthreshold PTSD diagnostic criteria (Hellmuth *et al.* 2012; Bovin *et al.* 2016; Dretsch *et al.* 2016). Based on this, we examined gene expression levels in soldiers using a dichotomized PCL-M score of less than (<) 34 and greater than or equal to (≥) 34.

Our findings are similar to that of Segman *et al.* (2005) in that gene expression signatures in the PBMCs of trauma-exposed survivors are informative of the later development of PTSD phenotype. We found expression levels of the LRP8 (low density lipoprotein receptor (LDL) receptor related protein 8, or ApoER2) gene, which is highly expressed in the hippocampus and amygdala, and GOLM1 (golgi membrane protein 1) gene to be significantly greater in soldiers reporting symptoms of PTSD. In contrast, we found CYP2C8 expression levels to be nominally significantly down-regulated in the symptom group compared to the control group.

Expression levels of both the uncharacterized LOC100132215 noncoding (nc) RNA gene and long intergenic non-protein coding RNA 943 (LINC00943) were increased in the symptom group compared to controls. Although LOC100132215 has been shown to be expressed in many tissue types including the hippocampus and prefrontal cortex in the brain, the function has yet to be characterized (GTEx Consortium 2013). Similarly with LINC00943; expression levels were found within testes, however its function is unknown. Although preliminary, our findings are in concordance with research in that psychological trauma can alter the function of genes, resulting in short-term and/or long-lasting effects on neuronal function, brain plasticity and behavioral adaptations to psychological stressors (Zannas *et al.* 2015; Segman *et al.* 2005; Rampp *et al.* 2014; Provencal and Binder 2015).

### LRP8

It is well known that the Learning and Memory pathway lies at the core of PTSD (Miller and Sadeh 2014; Kohannim *et al.* 2012; Pan *et al.* 2014; Bouter *et al.* 2014; Birnie *et al.* 2013; Donnelly *et al.* 2015; Telese *et al.* 2015). The pathways takes place in the brain and are dynamic processes; throughout life we are constantly acquiring new knowledge through *learning* and storing information through *memory*. Both processes are thought to be governed by synaptic plasticity (Telese *et al.* 2015; Mahan and Ressler 2012); synaptic connections are remodelled (changes in the neural circuitry level) based on environmental cues and the brain learns to recognize and remember danger through a process known as long-term potentiation (LTP). This process may last for months or longer and is essential for some forms of learning, the formation and retention of long-term memories, and synaptic plasticity (Mahan and Ressler 2012). The Reelin pathway has been well documented to regulate synaptic plasticity and memory formation in the brain (Telese *et al.* 2015; Lee and D’Arcangelo 2016; Li *et al.* 2003; Chen *et al.* 2010) and LRP8 serves as a classical signaling receptor for Reelin thereby mediating it’s effect (Telese *et al.* 2015; Lee and D’Arcangelo 2016; Li *et al.* 2003; Chen *et al.* 2010; Gilat-Frenkel *et al.* 2014; Beffert *et al.* 2005). LRP8 is abundantly expressed on neurons in the central nervous system (CNS). In fact, expression data from GTEX (using RNA-seq from 150 post-mortem donors with a total of 3,797 tissues samples) and the Human Brain Transcriptome database confirm that LRP8 is highly expressed in the hippocampus and amygdala (Li *et al.* 2016). Downstream effects include the activation of synaptic plasticity genes involved in the fear conditioning paradigm and memory formation. For example, in a mouse model of genetically manipulated inhibition of the Reelin/LRP8 signaling pathway, mice demonstrated marked fear-conditioning deficits and Learning disabilities (Beffert *et al.* 2005; Weeber *et al.* 2002). Moreover, the hippocampus and amygdala are two regions of the brain whose aberrant functions are associated with mental health disorders (Zimmerman *et al.* 2012; Segman *et al.* 2005). More specifically, a non-synonymous SNP rs5174 within LRP8 gene was found to be significantly associated with SCZ and BPD as well as higher LRP8 expression, suggesting the risk of SCZ and BPD is strongly associated with LRP8 (Li *et al.* 2016). On the other hand, mRNA expression levels of LRP8 within The peripheral blood lymphocytes in patients diagnosed with Major Depressive Disorder (MDD) were found to be lower compared to controls. In our study, we found expression levels of LRP8 to be significantly greater in soldiers reporting symptoms of PTSD. Although the findings suggest that LRP8 levels may serve as a peripheral biomarker, the authors did not find any correlation between expression levels and severity of depression symptoms (Suzuki *et al.* 2010). At the very least, it supports that the LRP8 signaling pathway may play a role within the pathophysiology of this mental health disorder as well.

LRP8 or Apolipoprotein (ApoE) is also known to maintain cholesterol homeostasis, which is critically important for brain function, *i.e.*, neuronal physiology such as, synapse formation and development (Zhang and Liu 2015; Petrov *et al.* 2016). In addition, isoforms E2 and E4 have been shown to promote atherosclerosis via Reelin by increasing vascular inflammation (Ding *et al.* 2016), thereby increasing the risk for cerebrovascular and cardiovascular pathologies (Lopez *et al.* 2014) as well as neurological pathologies, specifically Alzheimer’s disease (Weiner *et al.* 2014). Interestingly, the link between PTSD and an increased risk for cardiovascular disease (CVD) was discovered soon after the Civil War by Dr. Da Costa, who coined the term ‘soldier’s heart” or “irritable heart”. However, only in the last few years has genetic evidence linking PTSD with an increased risk CVD been emerging (Pollard *et al.* 2016; Guardado *et al.* 2016; Roy *et al.* 2015). In a candidate gene approach, Pollard *et al.* (2016) identified 106 PTSD studies that report one or more polymorphic variants in 87 candidate PTSD risk genes from 83,463 subjects and controls. Among their network analyses, using Ingenuity Pathway Analysis, the nuclear factor - κB (NF-κB) complex was identified as the principal hub for PTSD and CVD risk genes. This is not surprising, since NF-κB plays a key role in cellular stress response, governing the expression of many inflammatory genes (Li *et al.* 2002), a large number of which have been linked to PTSD (Feodorova and Sarafian 2012). ApoE came up as an influencing gene and one of the top 5 hubs for Type 2 Diabete Mellitus (Pollard *et al.* 2016). In fact, using genome-wide sequencing in a human ApoE3 and E4 mouse model, the authors found an Increase in the expression of inflammation-related genes by ApoE4 via greater activation of NF-κB Genes (Ophir *et al.* 2005). Numerous studies have linked PTSD with an elevation in peripheral inflammatory markers and inflammatory-related diseases (Lindqvist *et al.* 2017). These studies support our findings of an increase in the expression of the ApoE/LRP8 gene in soldiers reporting symptoms of PTSD.

### GOLM1

Recently there has been a lot of overlap in genes affecting the risk to developing aging-related Alzheimer’s Disease, Parkinson’s Disease and other neurodegenerative disorders, with that of PTSD (Kempuraj *et al.* 2017). This is not surprising considering that PTSD is well-documented to be a risk factors for Alzheimer’s Disease (Weiner *et al.* 2014; Lindqvist *et al.* 2017). One particular candidate gene for susceptibility, as it pertains to the findings in this study, is the Golgi membrane protein or golgi membrane protein 2 or GOLM1 gene (Kohannim *et al.* 2012). In our study, the expression of GOLM1 was found to be significantly increased in soldiers reporting symptoms of PTSD, compared to the control group. GOLM1plays a key role in processing proteins from the endoplasmic reticulum (ER) and subsequently transporting them (in vesicles) for release at the synapse. In a mouse model of aged Alzheimer’s disease, GOLM1 expression levels were significantly elevated (Bouter *et al.* 2014). This may be due to the fact that production of the beta amyloid (Aβ) peptide, which is associated with the onset of AD, is also associated with the endoplasmic reticulum (ER) and Golgi (Henriques *et al.* 2016). Aβ accumulation has been associated in humans with chronic stress as well as animal models of chronic stress, suggesting that immune disorders with excessive inflammatory reaction, such as PTSD, are key players in this neurodegenerative disorder (Kempuraj *et al.* 2017). Accelerated AD pathogenesis also involves an increase in phosphorylated tau levels, and levels in the cerebrospinal fluid have been shown to be directly influenced by the APOE locus in patients with AD (Deming *et al.* 2017).

An increase in oxidative stress is also associated with AD pathogenesis in conditions of chronic stress and abnormal inflammatory states (Kempuraj *et al.* 2017) and is known to be initiated or potentiated by traumatic stress such as PTSD (Miller and Sadeh 2014). Oxidative stress is known to induce a pro-inflammatory state from mast cells, as well as perturb the endoplasmic reticulum (ER), thereby affecting critical roles such as intracellular calcium homeostasis, lipid biosynthesis, protein folding and transport. Mast cells have been shown to be dysregulated in combat soldiers, thereby augmenting inflammation. This may explain the observed increase in peripheral inflammatory markers and total inflammatory scores in combat experienced veterans with PTSD, as compared to veterans without PTSD (Lindqvist *et al.* 2017). In our study population, soldiers reporting symptoms of PTSD also reported higher combat scores. ER stress has been associated with CVD, diabetes and neurodegenerative disorders. In fact, β-amyloid plaques were shown to induce ER stress (Sozen and Ozer 2017), suggesting Possible deposition in our soldiers reporting symptoms of PTSD. One may therefore hypothesize that an increase in the expression of GOLM1 is a reflection of high ER stress.

### CYP2C8

CYP2C8 is an enzyme and member of the cytochrome P450 (CYP) family. This enzyme plays an important role in metabolizing retinoic acid and arachidonic acid into epoxyeicosatrienoic acids (EETs) (Birnie *et al.* 2013; Donnelly *et al.* 2015). It is thought that EETs may improve cerebral blood flow and vascular tone in the brain after a brain injury (Donnelly *et al.* 2015). Liu *et al.* (2014) who found an overexpression of CYP2C in human umbilical vein endothethial cells exerted anti-oxidative and anti-vascular inflammatory effects via EETs (Liu *et al.* 2014). In an animal model using male Sprague-dawley rates, EETs were shown to exert anti-hypertensive properties (Khan *et al.* 2014). In our study, we found CYP2C8 expression levels to be significantly down-regulated in the symptom group compared to the control group, suggesting a compromised state of recovery or quite possibly an increased risk Of atherosclerosis (Liu *et al.* 2014) . Furthermore, knowing that there is strong association between PTSD and mild TBI in soldiers exposed to combat (Ruff *et al.* 2012), there is good reason to tag relevance to this gene warranting the collection of TBI history in future studies.

## Conclusion

Troops who have recently returned from tour and were involved in combat comprise a high-risk population group for developing PTSD. This pilot study is the first to report transcriptome-wide expression profiling reflecting PTSD endophenotype in peripheral blood samples from Canadian Armed Forces soldiers within one-year of returning from tour in Afghanistan. Using NGS, this study sought to characterize a molecular signature that defines risk of *PTSD – a complex psychiatric disorder*, from the peripheral blood of soldiers immediately returning within one-year from tour in Afghanistan. Soldiers reporting symptoms of PTSD (symptom group) also reported a trend toward higher combat scores compared to those in the control group. After correcting for confounding variables, a handful of top-genes were discovered to be differentially expressed in the Symptoms of PTSD group. We found the expression of genes LRP8 and GOLM1 were significantly upregulated and the expression of CYP2C8 to be significantly downregulated, compared to Control (no symptoms of PTSD). Collectively, these findings suggest the following: 1) perturbation in the hippocampus and amygdala, possibly due to experiencing a fearful event, thus leading to PTSD symptoms, 2) a possible decrease in cognitive reserve due to loss or compromised neural integrity, 3) the progression of early onset Alzheimer’s Disease, 4) a disruption in cholesterol homeostasis and quite possibly an increased risk of atherosclerosis and CVD - all of which has been shown in patients with PTSD.

Albeit preliminary, not only do these findings reflect a genomic predisposition to developing PTSD, but also demonstrate that gene expression signatures in PBMCs contain information reflective of symptoms of PTSD. As such, the results provide proof-of-principle for the potential diagnostic value of blood-based gene expression signatures for providing early insight prior to threshold defined clinical PTSD. Finally, the study highlights the use of genome information to point out risk of delayed diseases to human health and well-being. Further validation and replication using a larger sample size and GWAS dataset, is required for early prediction and thus focused early intervention.
